# An ethylene-induced NAC transcription factor acts as a multiple abiotic stress responsor in conifer

**DOI:** 10.1093/hr/uhad130

**Published:** 2023-06-20

**Authors:** Fangxu Han, Peiyi Wang, Xi Chen, Huanhuan Zhao, Qianya Zhu, Yitong Song, Yumeng Nie, Yue Li, Meina Guo, Shihui Niu

**Affiliations:** National Engineering Research Center of Tree Breeding and Ecological Restoration, College of Biological Sciences and Technology, Beijing Forestry University, Beijing 100083, China; National Engineering Research Center of Tree Breeding and Ecological Restoration, College of Biological Sciences and Technology, Beijing Forestry University, Beijing 100083, China; National Engineering Research Center of Tree Breeding and Ecological Restoration, College of Biological Sciences and Technology, Beijing Forestry University, Beijing 100083, China; National Engineering Research Center of Tree Breeding and Ecological Restoration, College of Biological Sciences and Technology, Beijing Forestry University, Beijing 100083, China; National Engineering Research Center of Tree Breeding and Ecological Restoration, College of Biological Sciences and Technology, Beijing Forestry University, Beijing 100083, China; National Engineering Research Center of Tree Breeding and Ecological Restoration, College of Biological Sciences and Technology, Beijing Forestry University, Beijing 100083, China; National Engineering Research Center of Tree Breeding and Ecological Restoration, College of Biological Sciences and Technology, Beijing Forestry University, Beijing 100083, China; National Engineering Research Center of Tree Breeding and Ecological Restoration, College of Biological Sciences and Technology, Beijing Forestry University, Beijing 100083, China; National Engineering Research Center of Tree Breeding and Ecological Restoration, College of Biological Sciences and Technology, Beijing Forestry University, Beijing 100083, China; National Engineering Research Center of Tree Breeding and Ecological Restoration, College of Biological Sciences and Technology, Beijing Forestry University, Beijing 100083, China

## Abstract

The proper response to various abiotic stresses is essential for plants' survival to overcome their sessile nature, especially for perennial trees with very long-life cycles. However, in conifers, the molecular mechanisms that coordinate multiple abiotic stress responses remain elusive. Here, the transcriptome response to various abiotic stresses like salt, cold, drought, heat shock and osmotic were systematically detected in *Pinus tabuliformis* (*P. tabuliformis*) seedlings. We found that four transcription factors were commonly induced by all tested stress treatments, while *PtNAC3* and *PtZFP30* were highly up-regulated and co-expressed. Unexpectedly, the exogenous hormone treatment assays and the content of the endogenous hormone indicates that the upregulation of *PtNAC3* and *PtZFP30* are mediated by ethylene. Time-course assay showed that the treatment by ethylene immediate precursor, 1-aminocyclopropane-1-carboxylic acid (ACC), activated the expression of *PtNAC3* and *PtZFP30* within 8 hours. We further confirm that the PtNAC3 can directly bind to the *PtZFP30* promoter region and form a cascade. Overexpression of *PtNAC3* enhanced unified abiotic stress tolerance without growth penalty in transgenic* Arabidopsis* and promoted reproductive success under abiotic stress by shortening the lifespan, suggesting it has great potential as a biological tool applied to plant breeding for abiotic stress tolerance. This study provides novel insights into the hub nodes of the abiotic stresses response network as well as the environmental adaptation mechanism in conifers, and provides a potential biofortification tool to enhance plant unified abiotic stress tolerance.

## Introduction

Global warming and extreme weather make abiotic stress as an important factor affecting world food security [1–3]. Plants grown in natural environments always face different survival stress. In addition, biotic stress like herbivorous animal attacks, human activities and diseases caused by microorganisms have certain effects on plants [4, 5]. In the context of global warming, the effects of abiotic stresses on plant growth and development become more pronounced [6–9].

Abiotic stress normally refers to adversity growth conditions like drought, salt, heat, and cold, as well as ultraviolet (UV) which can affect plant growth [10]. Since the Green Revolution, great efforts had been taken by scientists to enhance plant ability to resist abiotic stress and shorten plant lifespan without affecting yield [11]. It is a great challenge to enhance plant abiotic stress resistance without affecting its growth in plant biotechnology by the reason of the trade-off between defense and yield [12, 13].

To realize this ideal, the regulatory mechanisms of response to abiotic stress in model plants have been extensively studied. In angiosperm, *CPKs* and *MAPKs* family genes were regarded as responders that responded to multiple abiotic stress by the abscisic acid (ABA) signaling pathway [5]. To understand the abiotic stress regulatory mechanisms, the receptors and signaling pathways of ABA have been identified [14]. However, what the scene is in conifers remains elusive. Although some clues indicate ABA participates in some abiotic stress responses in conifer, some abiotic stress related genes had been identified [15–17]. The integration mechanism of multiple abiotic stress response pathways in conifer is still unknown.

Conifer serves as one of the most widely distributed trees in the northern hemisphere and has crucial ecological and economic value. As a gymnosperm, conifer accounts for 39% of the global forests and possesses 615 extant species due to its super adaptability [18]. For conifers, the ability to respond to abiotic stress not only predetermined potential distribution but also the length of the life-span. There was much evidence to prove conifers have a stronger ability against unfavorable environmental conditions [10, 16, 19]. Interestingly, the whole genome-wide analysis of *Pinus tabuliformis* (*P. tabuliformis*) found C-repeat/DREB binding factors (CBFs), which played an important role in abiotic stress in angiosperm, were lacking in conifer [19], indicating they probably have a different abiotic stress response pathway in conifer.

NAC transcription factors (TFs) especially the ATAF subfamily have been widely reported as stress-related TFs [20–22]. In rice, stress-related NAC genes, such as *OsNAC5, OsNAC6, OsNAC9*, and *OsNAC10* responded to drought, high salinity, and ABA [23]. Overexpressed *OsNAC6*, *OsNAC9,* and *OsNAC10* enhanced drought stress resistance and overexpressed *OsNAC5*, *OsNAC9,* and *OsNAC10* increased grain yield under both normal and drought conditions [24, 25]. Compared to the other three TFs, *OsNAC5* responded to almost all abiotic stress, overexpressed *OsNAC5* increased plants tolerance to multiple abiotic stress [26], and owing to its powerful function in abiotic stress response, *OsNAC5* was regarded as a potential tool for biofortification strategies in rice [27]. The NAC genes *RhNAC3* and *JUNGBRUNNEN1* (*JUB1*) were reported to enhance dehydration tolerance in rose [28] and drought tolerance in tomatoes [29]. Ectopic expression of *HaNAC1, VvNAC17, ZmSNAC13, PeNAC034, PeNAC045*, and *PeNAC036* in *Arabidopsis* enhanced multiple abiotic stresses tolerance in an ABA pathway-dependent manner [30–33]. Taken together, NAC TFs played a critical role in enhancing abiotic stress tolerance, and the function was conserved in angiosperm.

In gymnosperm, SNAC family members participated in abiotic stress response in many cases. In recent years, *PpNAC2* and *PpNAC3* were isolated in *Pinus pinaster* and were found in response to multiple abiotic stress like salt, cold, and wounding treatment [34]. In Norway spruce (*Picea abies*), *PaNAC03* was found induced by stress and participated in stress defense by secondary metabolite production [35]. In *Picea wilsonii*, *PwNAC2* was reported to enhance plant tolerance under abiotic stress [36]. In *P. tabuliformis*, *PtNAC3* was found to be related to cold stress [37]. Although part of the SNAC family gene was found related to abiotic stress, there were 26 members in *P. tabuliformis*, and their role in adversity requires more research.

In conifers, the molecular mechanisms that coordinate multiple abiotic stress responses remain largely unknown. Here, we systematically detected the transcriptomic response to various abiotic stresses like salt, cold, drought, osmotic, and heat shock in *P. tabuliformis* seedlings. The core TFs that respond to multiple abiotic stresses were selected, and a NAC family member was highly induced by all test treatments. Furthermore, overexpression of the *PtNAC3* enhanced unified abiotic stress tolerance without growth penalty in transgenic *Arabidopsis*. Interestingly, we show that the activation of *PtNAC3* was mediated by ethylene (ET) rather than ABA. These results provide novel insights into the hub nodes of the abiotic stresses response network as well as the environmental adaptation mechanism in conifers, and provide a potential genetic tool to enhance plant unified abiotic stress tolerance.

## Results

### The transcriptomic landscapes and hub genes response to diverse abiotic stress in *P. tabuliformis*

**Figure 1:**
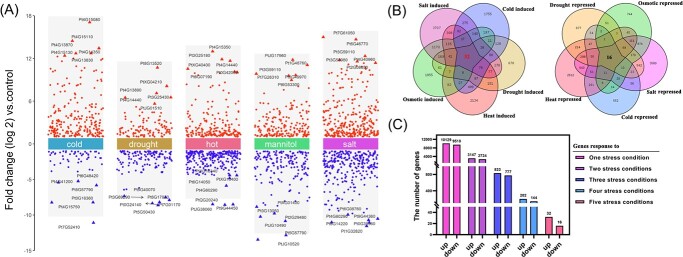
The response profiles of global transcriptome and commonly induced genes under different abiotic stresses. **A** Differential TFs expression analysis showing up- and down-regulated TFs across abiotic stress treatment. The *Pinus tabuliformis* seedlings were treated under five different abiotic stress, such as cold (1-month-old seedlings were treated under 4°C for 8 h), drought (3-year-old seedlings were treated without water for 23 days), heat (1-month-old seedlings were treated under 40°C for 8 h), salt (2-month-old seedlings were treated under 2 M NaCl for 3 days) and osmotic (2-month-old seedlings were treated under 300 mM mannitol for 3 days); needles were harvested for RNA-seq. The abiotic stresses driving TFs were harvested after differential expression analysis toward RNA-seq via edge R. **B** The Venn diagram analysis was conducted to determine the differential genes expressed in *P. tabuliformis* needles under five different abiotic stresses. **C** The histogram displays the number of abiotic stress induced/repressed genes in response to abiotic stress. The dates were visualized by GraphPad.

Conifers are adaptive to harsh environments, such as extreme cold and drought, particularly many species in the pine family have large distribution areas covering multiple climate zones in the northern hemisphere [2]. To investigate the core genes response to different abiotic stress in conifer, the seedlings of *P. tabuliformis* were treated with cold (4°C), heat (40°C), drought, salt (2 M NaCl), and osmotic (300 mM mannitol). RNA-seq analysis showed that different stress treatments tended to induce specific transcriptome response profiles, such as, 3222/3137 genes were specifically induced/repressed by salt, 1760/1824 genes were specifically induced/repressed by heat, 1244/510 genes were specifically induced/repressed by osmotic, 1209/341 genes were specifically induced/repressed by cold, 532/692 genes were specifically induced/repressed by progressive drought. Meanwhile, the most significant differential gene induced/repressed by different abiotic stress shared less similarity (Fig. 1A; Fig. S1A, see online supplementary material). Strikingly, 32/16 induced/repressed genes were shared by all abiotic stress treatments (Fig. 1B and C). The 32 abiotic stress induced genes (ASIG) include four TFs: *PtNAC3*, *PtNAC5*, *PtMYB175* and *PtZFP30* (Table S2, see online supplementary material). The expression profiling showed that NAC (NAM/ATAF/CUC) transcription factors *PtNAC3* and *PtNAC5* have higher expression levels and more obvious differences than the other two TFs when suffering abiotic stress (Fig. S1B, see online supplementary material), indicating NAC TFs may play an important role in the abiotic stress response of conifers.

### *PtNAC3* acts as a core responsor of abiotic stresses response in *P. tabuliformis*

**Figure 2:**
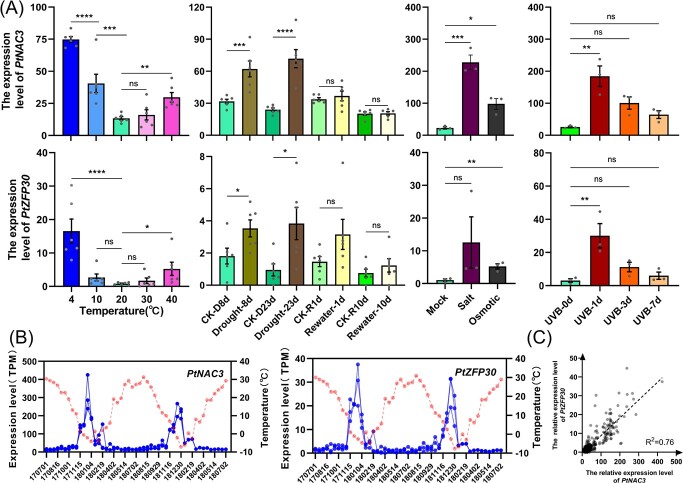
The expression pattern of abiotic stress-induced transcription factors *PtNAC3* and PtZFP30. **A** The transcript levels of *PtNAC3* and *PtZFP30* induced by different abiotic stress. The first column represents the expression level of *PtNAC3* and *PtZFP30* under different temperatures. 1-month-old *Pinus tabuliformis* seedlings were treated with five temperature gradients (4°C, 10°C, 20°C, 30°C, and 40°C) for 8 hours under long-day (14 h/10 h), and the needles were harvested for RNA-seq. Data are shown as mean ± SEM (*n* = 6). The second column represents the expression level of *PtNAC3* and *PtZFP30* under drought. Three-year-old seedlings were treated without water for 8 or 23 days and then rewatered for 1 or 10 days then harvested the needles for RNA-seq. Data are shown as mean ± SEM (*n* = 6). The third column represents the expression level of *PtNAC3* and *PtZFP30* under salt and osmotic treatment. Two-month-old seedlings were treated under long-day (14 h/10 h) and irrigated with 2 M NaCl or 300 mM mannitol once a day for 3 days, then harvested needles for RNA-seq. Data are shown as mean ± SEM (*n* = 3). The fourth column represents the expression level of PtNAC3 and PtZFP30 under UVB. Seedlings of 2.5 years old were irradiated by ultraviolet radiation for 1, 3, or 7 days in the greenhouse before being harvested for RNA-seq. Data are shown as mean ± SEM (*n* = 6). Statistics, *t* tests (^*^P < 0.05, ^**^P < 0.01, ^***^P < 0.001, ^****^P < 0.0001). **B** The annual expression level of abiotic stress induced transcription factors (ASITF) *PtNAC3* and *PtZFP30* in *P. tabuliformis* from 1 July 2017 to 30 December 2018 in Beijing. The X-axis indicates the sampling date from 1 July 2017 to 2 July 2018, the left Y-axis indicate the transcript levels of *PtNAC3* and *PtZFP30*, the right-Y-axis indicate the average temperature on that day. The needles were collected from three individual trees at 11 a.m. The date was visualized by GraphPad. Data are shown as mean ± SEM (*n* = 3). **C** The co-expression relativity between *PtNAC3* and *PtZFP30* in *P. tabuliformis* under abiotic stress. The transcript date of *PtNAC3* and *PtZFP30* shown in Table S2 (see online supplementary material) under abiotic stress treatment were used to analyse correlation coefficients. GraphPad was used to calculate the correlation coefficients with default parameters.

We further analysed the detailed expression profiles of these multiple stress response TFs under moderate unfavorable growing conditions (Fig. 2; Fig. S2, see online supplementary material). All four abiotic stress-induced TFs show a similar response profile to these stress conditions; however, compared with other ASIGs, *PtNAC3* showed a more sensitive and vigorous expression response, indicating that it probably acts as a core transcription activator in abiotic stress response. The result showed that *PtNAC3* was up-regulated under 4°C, 10°C, 30°C, 40°C temperatures compared with 20°C, and induced by 8 days of moderate drought treatment and slightly up-regulated for 23 days of progressive drought treatment, while its expression level was recovered to control level after rewatering one day [16]. We found that the response of these genes should be a very rapid process. For example, one day of UVB treatment is enough to induce the expression of *PtNAC3* hundreds of times, and 8 hours of injury treatment can activate its response [10]. The *PtZFP30* (*PtJG40760*), *PtNAC5* (*Pt2G26090*), and *PtMYB175* (*Pt3G25430*) also show a similar response profile albeit to a lesser extent. Compared to other treatments, including osmotic stress induced by mannitol, NaCl treatment induced the expression of these genes to the greatest extent (Fig. 2A; Fig. S2, see online supplementary material).

As stress response genes, the four TFs were expressed at low levels during normal growth seasons in the field during the annual cycle, but only highly accumulate in the winter (Fig. 2B, Fig. S2, Fig. S6, see online supplementary material). In the diurnal cycle, these four TFs were also only showing high accumulation in winter and did not show obvious circadian rhythm (Fig. S2, see online supplementary material). Notably, the expression patterns of these four TFs were very similar, and the correlation coefficient between the expression of *PtZFP30* and *PtNAC3* was R^2^ = 0.76 (Fig. 2C), indicating that they may be in the same response pathway.

### PtNAC3 is a stress-related NAC (SNAC) transcription factor

**Figure 3:**
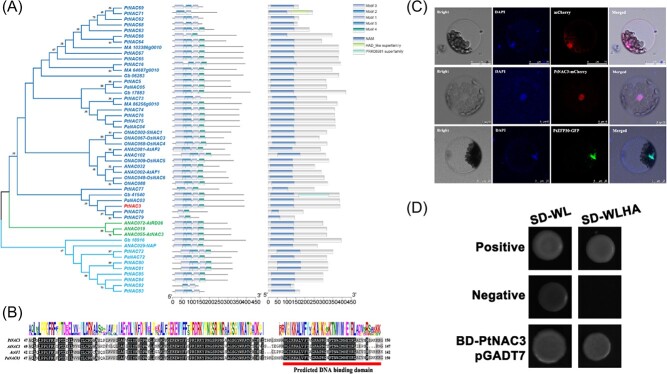
PtNAC3 is a conserved stress-related NAC (SNAC) transcription factor. **A** Phylogenetic analysis of the motif pattern and conserved domain analysis of ATAF/SNAC sub-family proteins from different species. The full-length protein sequences from *Arabidopsis thaliana, Oryza sativa, Ginkgo biloba, Picea abies* and *Selaginella moellendorffii *were used to build the tree. The supporting values are shown on the branches in the following order: SH-aLRT test/bootstrap value. **B** Multiple-sequence alignment of *PtNAC3, AtNAC3, AtAF1* and *PaNAC03* protein sequences in *Pinus tabuliformis*. The conserved amino acids were shaded, predicted DNA binding domain was marked by the bar. Sequence alignment analyses were conducted in DNAMAN 8. **C** Subcellular localization of PtNAC3 and PtZFP30. To analyse the subcellular of PtNAC3 and PtZFP30, the vector of *35S-PtNAC3-mCherry* and *PtZFP30-GFP* were transformed into the protoplasts of *A. thaliana* leaf and *35S-mCherry* were transformed as a control. After overnight culture, the transformed protoplasts were stained with 10 μg/mL DAPI for 30 minutes before being observed using a Leica Scanning microscope. Bar = 10 μm. **D** Y2H assays of transcriptional activation of PtNAC3. Yeast cells co-transformed with BD-PtNAC3/AD-T7 were grown on selective media (SD/−Trp-Leu-His-Ade). AD-T and BD-p53 were used as positive control; AD-T and BD-Lam were used as negative control.

The NAC genes belong to a large TF family in *P. tabuliformis*, which includes 123 members in the genome (Fig. S3, Table S3, see online supplementary material). By the phylogenetic analysis of the NAC family in *P. tabuliformis*, both PtNAC3 and PtNAC5 belong to the SNAC/ATAF subfamily (Fig. S3, see online supplementary material), which has been widely reported to participate in abiotic stress in plants [20, 21]. PtNAC3 has a typically conserved N-terminal NAC domain (Fig. 3A), in which the amino acid residues that are critical for DNA binding ability are identical with the ATAF1 and AtNAC3 in *Arabidopsis* (Fig. 3B). Consistent with its transcription factor function, the PtNAC3 protein was exclusively located in the nucleus of *Arabidopsis* protoplasts (Fig. 3C), and can activate the GAL4 reporter gene transcription in yeast (Fig. 3D).

### PtNAC3 positively regulates *PtZFP30* by directly binding its promoter

**Figure 4:**
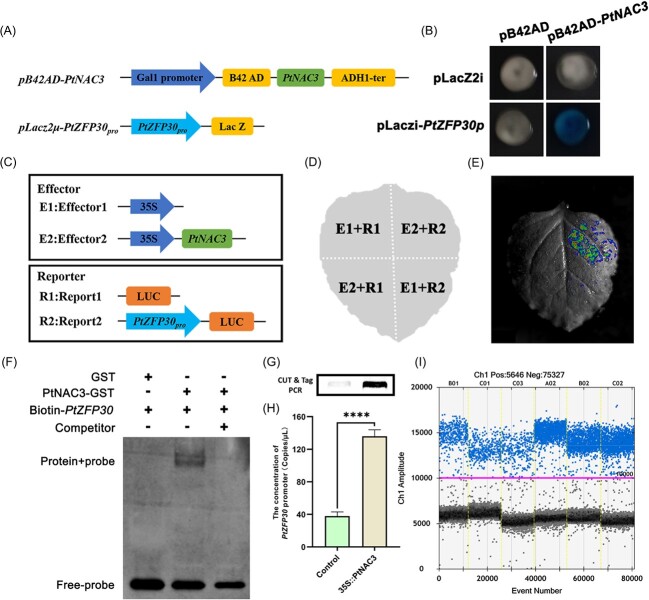
*PtNAC3* positively regulates *PtZFP30* by directly binding the promotor of *PtZFP30*. **A** Schematic of pB42AD-*PtNAC3* and pLacz2μ-*PtZFP30* promoter vectors for Y1H assay. The promoter of *PtZFP30* fused the pLacZ2μ vector, the *PtNAC3* gene was fused to the pB42AD vector, pLacZ2μ vector and pB42AD vector was used as the negative control. **B** Y1H assay of PtNAC3 directly binding the promoter of *PtZFP30*. Yeast cells co-transformed with pLacz2μ-*PtZFP30*/pB42AD-*PtNAC3* were grown on selective media (SD/−Trp/-Ura + Gal+Raf + X-gal). pLacZ2μ/pB42AD, pLacZ2μ/pB42AD-*PtNAC3,* pLacz2μ-*PtZFP30*/pB42AD were used as negative controls. **C**–**D** Schematic of the effector and reporter structures for *PtNAC3* (E2) and *PtZFP30* promoter (R2). **E***PtNAC3* positively regulates *PtZFP30*. LUC assays for detecting PtNAC3*-PtZFP30* promoter interactions were performed in *Nicotiana benthamiana* leaves. The *Agrobacterium tumefaciens* GV3101 cells which have recombinant plasmid shown in **C** were equal volume mixed and infiltrated into *N. benthamiana* leaves. The leaves were sprayed with D-Luciferin potassium salt and used for fluorescence detection by the LB983 NightOwl II. **F** EMSA assays were applied to identify the interactions between GST-PtNAC3 protein and the *PtZFP30* promoter. **G**–**I** A transient transformation system to transiently express *35S::PtNAC3-HA-GUS* and *35S::HA-GUS* as a negative control in the hypocotyl of *Pinus tabuliformis*, CUT & Tag assays were applied to identify the interactions between PtNAC3-HA protein and the *PtZFP30* promoter in *P. tabuliformis*. **G** After obtaining the CUT & Tag library, 2 μL libraries were diluted in 8 μL ddH_2_O as templates to run PCR. Primers were listed in Table S1 (see online supplementary material). **H**–**I** Droplet digital PCR was used to further confirm that PtNAC3 can bind the *PtZFP30* promoter *in vivo*.

*PtZFP30* belongs to the C2H2 family, which also has been reported to participate in abiotic stress response in plants [38] (Fig. S5, Fig. S7, see online supplementary material). By co-expression analysis, we found the *PtNAC3* co-expression TF *PtZFP30* has a putative AtNAC3 binding motif in its promoter region [39], indicating *PtNAC3* may directly target the *PtZFP30* and form a cascade in the stress response pathway. To investigate this possibility, we used the yeast one-hybrid (Y1H) assay [40], dual-luciferase reporter assay [41], electrophoretic mobility shift assay (EMSA) and CUT & Tag assay to test the role of PtNAC3 in the regulation of *PtZFP30*. The results showed that PtNAC3 binds to the *PtZFP30* promoter in yeast and digested X-gal and let yeast turn blue (Fig. 4A and B). To confirm whether PtNAC3 regulated the expression of the *PtZFP30*, we use *Nicotiana benthamiana* leaves to run a transient expression assay (Fig. 4C and D). The result confirmed that PtNAC3 could activate the *PtZFP30* promoter activity (Fig. 4E). Moreover, EMSA confirmed that PtNAC3 binds to the promoter of *PtZFP30* containing the ACACGTAA motifs (Fig. 4F). An *in vivo* study was performed by a transient transformation system to transiently express *35S::PtNAC3-HA-GUS* and *35S:: HA-GUS* in the hypocotyl of *P. tabuliformis.* With CUT & Tag assay, the results of PCR and droplet digital PCR showed that PtNAC3 binds the promoter of *PtZFP30* occurred in *P. tabuliformis* (G–I). Taken together, these results indicate that PtNAC3 serves as the core responsor in the upstream of *PtZFP30* in the abiotic stresses response pathway in *P. tabuliformis*.

### The induction of *PtNAC3* was associated with the ethylene pathway under abiotic stress

**Figure 5:**
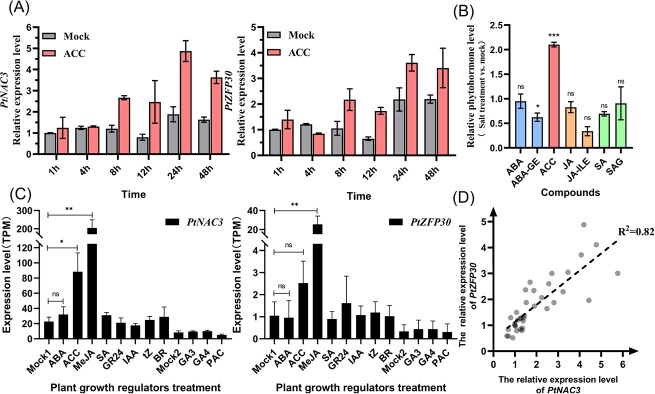
The ethylene associated with *PtNAC3* and *PtZFP30* expression under abiotic stress. **A** Enhancing the expression level of *PtNAC3* and *PtZFP30* under the abiotic stress of ethylene. Two-month-old *Pinus tabuliformis* seedlings were sprayed daily with common plant growth regulators (100 μM ABA, 2 mM ACC, 50 mM MeJA, 1 mM SA, 300 μM GR24, 100 μM IAA, 10 μM tZ,10 μM BR, 50 μM GA_3_, 50 μM GA_4 + 7_, 50 μM PAC) under long-day (14 h/10 h) for 3 days, needles were harvested for RNA-seq analysis. Data were shown as mean ± SEM (*n* = 3). Statistics, *t* tests (^*^P < 0.05, ^**^P < 0.01). **B** Measurement of phytohormones level in the needles under abiotic stress of salt. Two-month-old *P. tabuliformis* seedlings were treated with 2 M NaCl three times under long-day (14 h/10 h) for two weeks, needles were collected and sent to MetWare based on the AB Sciex QTRAP 6500 LC–MS/MS platform to detect phytohormones contents. ABA, abscisic acid; ABA-GE, ABA-glucosyl ester; ACC, 1-Aminocyclopropanecarboxylic acid; JA, jasmonic acid; JA-ILE, Jasmonoyl-L-isoleucine; SA, salicylic acid; SAG, salicylic acid 2-O-β-glucoside. The date was visualized by GraphPad. Data are shown as mean ± SEM (*n* = 3). Statistics, *t* tests (^*^P < 0.05, ^***^P < 0.001). **C** Relative expression of *PtNAC3* and *PtZFP30* under the treatment of ACC. Two-month-old *P. tabuliformis* seedlings were treated by 2 mM ACC under long-day (14 h/10 h), after 1, 4, 8, 12, 24, 48 hours of treatment. Total RNA was isolated from needles for RT-qPCR analysis. The gene expression level in Mock was set as 1. The RT-qPCR primers are listed in Table S1 (see online supplementary material). Error bars are SD (*n* = 3). Statistics, *t* tests (^**^P < 0.01, ^***^P < 0.001). **D** The co-expression relativity between *PtNAC3* and *PtZFP30* in *P. tabuliformis* phytohormone treatment. The transcript date of *PtNAC3* and *PtZFP30* under phytohormone treatment shown in Table S2 (see online supplementary material) were used to analyse correlation coefficients. GraphPad was used to calculate the correlation coefficients with default parameters.

Phytohormones, especially for ABA-signaling pathways, have been well studied for their involvement in abiotic stress responses. To determine whether there was any connection between phytohormones and *PtNAC3-PtZFP30 *abiotic stress response pathway, 2-month-old *P. tabuliformis* seedlings were treated with ABA, 1-amino cyclopropane carboxylic acid (ACC), methyl jasmonate (MeJA), salicylic acid (SA), strigolactones (GR24), indole-3-acetic acid (IAA), trans-zeatin (TZ), brassinolide (BR), gibberellin A3 (GA_3_), gibberellin A4 and A7 (GA_4 + 7_), and paclobutrazol (PAC). Based on RNA-seq analysis, the expression level of both *PtNAC3* and *PtZFP30* was significantly upregulated under ACC and MeJA treatment, but not response to ABA (Fig. 5A). As the *PtNAC3* had the highest expression response under the salt treatment condition (Fig. 2A), we then measured the endogenous hormone levels in *P. tabuliformis* seedlings which were treated with 2 M NaCl for 3 days. Compared with the control condition, ACC was significantly accumulated, but there were no significant differences in ABA, ABA-GE, SA, and SAG, the JA content was also not changed; however, the JA active form JA-ILE level was significantly decreased (Fig. 5B). To confirm the ACC response and determine the response time of *PtNAC3* and *PtZFP30*, the time-course assay was conducted and showed that both *PtNAC3* and *PtZFP30* were significantly induced by ACC within 8 hours and the effect lasted until 48 hours (Fig. 5C). Under ACC treatment, *PtNAC3* and *PtZFP30* also tightly co-expressed with the R^2^ = 0.82 (Fig. 5D).

### *PtNAC3* confers tolerance to multiple abiotic stresses and promotes reproductive success by shortening the lifespan in *Arabidopsis*

**Figure 6:**
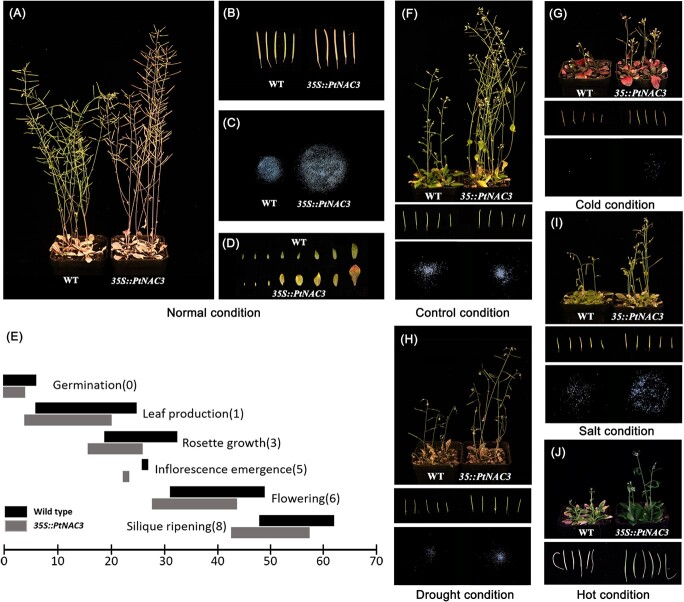
Ectopic expression of *PtNAC3* enhanced abiotic stress resistance and increased yields in transgenic *Arabidopsis.* (**A**–**D**) Phenotypic performance of *PtNAC3* in transgenic *Arabidopsis.* Scale bars, 1 cm*.***A** Ectopic expression of the *PtNAC3* gene (*35S::PtNAC3*) significantly grew faster than wild-type *Arabidopsis* (WT) after 8 weeks growth under long-day (16 h light/ 8 h dark) in the greenhouse. **B** The siliques from *35S::PtNAC3* were larger and senescence faster than that of WT after 8 weeks of growth under long-day (16 h light/ 8 h dark) in the greenhouse. **C** The total amount of seeds from *35S::PtNAC3* was more than WT after 8 weeks of growth under long-day (16 h light/ 8 h dark) in the greenhouse. **D** The leaf from *35S::PtNAC3* was senescence faster than WT after 8 weeks of growth under long-day (16 h light/ 8 h dark) in the greenhouse. **E** Schematic of the lifespan of transgenic line *35S::PtNAC3* and wildtype *Arabidopsis.* The *Arabidopsis* lifespan was recorded and transgenic line *35S::PtNAC3* was shorter than WT after 8 weeks of growth under long-day (16 h light/ 8 h dark) in the greenhouse. The black frame indicates wildtype *Arabidopsis* and the grey frame indicates transgenic line *35S::PtNAC3.***F**–**H** Phenotypic performance of *PtNAC3* in transgenic *Arabidopsis* under different abiotic stresses. Scale bars, 1 cm. **F** Under normal culture the transgenic line *35S::PtNAC3* grew faster and has more siliques than WT after 6 weeks of growth under long-day (16 h light/8 h dark) in the greenhouse. **G** Four-week-old *Arabidopsis* grew in the greenhouse and were transformed to cold treatment (4°C), *35S::PtNAC3* grew faster, had more siliques and higher yield than WT after cold treatment for two weeks. **H** Four-week-old *Arabidopsis* grown in the greenhouse were transformed to drought treatment (without water), *35S::PtNAC3* grew faster, had more siliques and higher yield than WT after drought treatment for two weeks. **I** Four-week-old *Arabidopsis* grew in the greenhouse and were transformed to salt treatment (200 mM NaCl), *35S::PtNAC3* grew faster, had more siliques and higher yield than WT after salt treatment for two weeks. **J** Four-week-old *Arabidopsis* grew in the greenhouse and was transformed to hot treatment (40°C), *35S::PtNAC3* grew faster, and had more siliques than WT after hot treatment for a week.

As the *PtNAC3* acts as a multiple abiotic stress response responder in *P. tabuliformis*, we were interested in whether it could be used as a biological tool that applied to plant breeding for unified abiotic stress tolerance. To investigate this possibility, we overexpressed *PtNAC3* in *Arabidopsis thaliana* (Fig. S4, see online supplementary material). Unexpectedly, *35S:PtNAC3* overexpression lines (OEs) did not exhibit any typical negative traits like growth retardation and lower reproductive yields phenotype of overexpressing stress response TFs under normal conditions. On the contrary, compared with wild-type (WT), OEs were more vigorous, such as growing faster and higher (Fig. 6A), having more and longer siliques (Fig. 6A and B) and higher yield (Fig. 6C). The OEs also showed early maturity traits by shortening lifespan (Fig. 6A, D and E), both leaf (Fig. 6B) and siliques (Fig. 6D) senescence faster in OEs than WT. Moreover, OEs grow faster and stronger than WT under both normal and abiotic stress conditions (Fig. 6F–J), and promote reproductive success under all tested stress treatment including drought (Fig. 6H), cold (Fig. 6G), salt (Fig. 6I), and hot (Fig. 6J). After 14 days without water treatment, OEs grow faster and stronger than WT, and OEs siliques are less affected by drought while the yields of OEs are higher than WT (Fig. 6H). After 14 days of 4°C treatment, both OEs and WT growth were affected, but OEs flowered earlier and had more siliques compared with WT (Fig. 6G). After 14 days of 200 mM NaCl treatment, the siliques of WT were about to fall and seeds fail to develop normally, while OEs were less affected and harvested more seeds (Fig. 6I). After 7 days of 40°C treatment, the siliques of WT were about to fall and seeds fail to develop normally, while OEs were less affected (Fig. 6J). These results suggest that *PtNAC3* has great potentialas a biological tool in transgenic plants and confers tolerance to various abiotic stresses without growth penalty.

## Discussion

Since the Green Revolution, many efforts had been taken to improve the crop's tolerance to stress [42]. With the increase of the greenhouse effect, abiotic stress had received more attention [42, 43]. The core regulatory pathways of plants' abiotic stress response in angiosperm were elucidated [4, 44]. As widely distributed species in the northern hemisphere, however, the molecular mechanism of conifer response to abiotic stress is largely unknown. In the present study, we identified 32/16 genes induced/repressed by all five common abiotic stress which includes four/two TFs (Fig. 1B and C). In the study of model plants, it was found that *CPKs* and *MAPKs* family genes play a crucial role in response to multiple abiotic stress [5, 45], but we observed it is TFs instead of protein kinases that participate in abiotic stress response in *P. tabuliformis*. NAC, MYB as well as C2H2 transcription factors were widely reported in responses to environmental pressure in plants [38, 43, 46]; in this study we found *PtNAC3* induced by all common abiotic stress more obviously than other TFs (Fig. S1, see online supplementary material), implying *PtNAC3* is important in abiotic stress response. In recent years, the C2H2-type zinc finger protein family was also identified in response to abiotic stress, *PeSTZ1* was reported to enhance freezing tolerance in *Populus euphratica* [38]; *SlZF3* has been reported to enhance salt tolerance in tomato [47]; *ZFP183* was reported involved in abscisic acid induced abiotic stress defense [48]. *PtZFP30*, a member of the ZFP transcription factor family, may play an important role in abiotic stress response.

Previous studies found ASITFs responded to abiotic stress sensitively [21, 49, 50]. In the study, we found *PtNAC3* up-regulates under drought treatment and down-regulates to a common level after rewatering a day. This interesting pattern also disappeared under UVB treatment: *PtNAC3* sharply up-regulates and gradually down-regulates (Fig. 2A). In the annual period, *PtNAC3* and *PtZFP30* up-regulate significantly in winter, because the winter in Beijing is extremely cold with drought all day long (Fig. 2B). The results suggested that *PtNAC3* and *PtZFP30* are important for *P. tabuliformis* to adapt to winter. However, they can also respond to multiple abiotic stresses. Combined with the data of daily periodicity, we found *PtNAC3* responded to abiotic stress very rapidly, but when abiotic stress disappeared, the expression profile of *PtNAC3* will immediately recover normally (Fig. S2, see online supplementary material). Although the expression level of *PtZFP30* is much lower than *PtNAC3* when plants suffer abiotic stress, *PtZFP30* showed a strong correlation with *PtNAC3* under abiotic stress by co-expression analysis (Fig. 2C). All the features mentioned above illustrate *PtNAC3* was sensitive to abiotic stress, and was suitable for being a bio-marker to check whether *P. tabuliformis* is under stress. Although there are many homologous genes in SNAC group TFs in *P. tabuliformis* (Fig. 3A), only *PtNAC3* and *PtNAC5* were found to respond to multiple abiotic stress. The function of other TFs needs further research.

In the past few years, genes belonging to the SNAC sub-family have been discovered, most of them were nucleic localization and acted as transcription activators [28, 31, 51]. In this study, we found *PtNAC3* is nucleic localization (Fig. 3C), implying the *NAC3-ZFP30* module may work in the nucleus; our result also showed *PtNAC3* activates the GAL4 reporter gene in yeast (Fig. 3D), indicating *PtNAC3* may activate *PtZFP30*. SNAC TFs had been reported to respond to abiotic stress in many cases; however, the downstream genes of SNAC TFs were less reported. In the study, Y1H assay, dual-luciferase assay, CUT & Tag and EMSA were performed to prove *PtNAC3* can bind the promoter of *PtZFP30* (Fig. 4), suggesting *PtZFP30* is one of the downstream genes of *PtNAC3*.

Hormones, especially ABA and JA, participate in many abiotic stress response pathways, and attention had been paid to clarifying the regulatory mechanism of ABA and JA pathways in abiotic stress [52–54]. In angiosperm, SNAC group TFs participated in abiotic stress signaling pathway dependent on ABA [55]; however, since being treated with various hormones, we found that *PtNAC3* and *PtZFP30* responded only to ACC and MeJA treatment, while *PtNAC3* and *PtZFP30* showed the most obvious response under salt stress. Therefore, we chose salt stress as a representative to verify the hormone content in *P. tabuliformis* under abiotic stress. Under salt treatment, the content of ABA in *P. tabuliformis* was stable, but ACC accumulated significantly. After ACC treatment for 8 hours, *PtNAC3* and *PtZFP30* began to be up-regulated until 48 hours (Fig. 5). These results showed that ACC, but not ABA, was involved in the *NAC3-ZFP30-*mediated abiotic stress signaling pathway in *P. tabuliformis*. However, whether such hormonal changes are similar under other abiotic stresses deserves further investigation. Our data provide valuable information for understanding the molecular mechanism of abiotic stress response in conifers. The results suggested that the *NAC3-ZFP30* module acting as a responder response to multiple abiotic stress was ethylene-induced and provided new insight into the study of ancient plants' response to abiotic stress.

In the study, we also found *PtNAC3* has great potential to be a biological tool in crop breeding. Previous studies found many SNAC group TFs overexpressed in plants not only enhanced plants' abiotic stress tolerance but also improved yield [26, 56, 57]. In some studies, SNAC group TFs also reported related to leaf senescence [40, 43, 58]. Our study suggested overexpressed *PtNAC3* in *Arabidopsis* enhanced abiotic stress tolerance, improved yield as well as promoted leaf senescence (Fig. 6). *PtNAC3* overexpressed plants seem to gather all the advantages SNAC group TFs had reported, which is suitable for being a biological tool.

## Materials and methods

### Plant materials and treatments

The *P. tabuliformis* seedlings were planted in 6 cm pots with turfy soil (PINDSTRUP) and grew in the greenhouse (16 h light/8 h night; 21 ± 1°C) for two months. The ecotype Columbia *Arabidopsis* (WT) and *35S::PtNAC3* transgenic lines were planted in pots with the turfy soil mixed with the nutrient soil Fangjie (2:1 v/v). *N. benthamiana* was planted in pots containing turfy soil mixed with vermiculite (1.2:1 v/v).

For temperature treatment, two-month-old *P. tabuliformis* seedlings were transformed to a 4°C, 10°C, 20°C, 30°C, and 40°C climate chamber for 8 hours during the light. Drought treatment raw data was from Tariq Pervaiz's study [16]; each treatment mentioned above has six repeats. For salt and osmotic treatment, *P. tabuliformis* seedlings were irrigated daily with 2 M NaCl or 300 mM mannitol for 3 days; UV treatment raw data was from Xu J's study [10]. For plant growth regulator treatment, 2-month-old *P. tabuliformis* seedlings were sprayed daily with 100 μM ABA, 2 mM ACC, 250 μM MeJA, 1 mM SA, 300 μM GR24, 100 μM IAA, 10 μM TZ, 10 μM BR, 50 μM GA_3_, 50 μM GA_4 + 7_, and 50 μM PAC for 3 days, each treatment has three repeats. After the treatment mentioned above, 2 g needles were collected.

For abiotic stress treatment of *Arabidopsis*, the WT and *35S::PtNAC3* grew in the greenhouse for 4 weeks before flowering, and then treatments were started; for salt treatment, 1 L 200 mM NaCl was watered in a tray twice a week for two weeks; for drought, treatment kept the tray without water for two weeks; for cold treatment, transfer the *Arabidopsis* to a cold treatment greenhouse (16 h light/8 h night; 4 ± 1°C) for two weeks; for hot treatment, transfer the Arabidopsis to a cold treatment greenhouse (16 h light/8 h night; 40 + 1°C) for a week.

### DNA/RNA extractions and cDNA synthesis

*P. tabuliformis* genomic DNA was extracted from the needles following the protocol of the Plant Genomic DNA kit (TIANGEN, Beijing, China). Total RNA from different tissues of *P. tabuliformis* or *Arabidopsis* was extracted following the protocol of the Vazyme Plant Total RNA Isolation Kit (Vazyme, Nanjing, China) and stored at −80°C. 1 μg total RNA was used for first-strand cDNA samples generated by the Vazyme kit (Vazyme, Nanjing, China).

### RNA-sequencing, qRT-PCR, detection of phytohormones, and bioinformatics analysis

The RNA-seq analysis followed the protocol of [37, 59]. The clean reads were mapped to the *P. tabuliformis* reference genome [19] and the transcript abundances were calculated by Kallisto software [60]. The RNA-seq data used in this study were listed in Table S2 (see online supplementary material).

The qRT-PCR was performed by QuantStudio 6 Rex (Thermo Fisher Scientific) with the protocol of TSINGKE qPCR Mix. Primer Primer5 (www.PremierBiosoft.com) was used to develop the primers listed in Table S1 (see online supplementary material).

The phytohormones contents were detected by LC–MS/MS. After 2 M NaCl was irrigated daily for 3 days, 2 g needles were collected from 2-month-old seedlings. Rapid grinding of samples into powder was performed by liquid nitrogen freezing, 50 mg samples were used and extractant added containing 75% methanol and 5% formic acid. After 12 000 *g*, 5 min centrifugation at 4°C, the supernatant was removed. The precipitate was re-dissolved by 100 μL 80% methanol and passed through the 0.22 μm filter and then injected vial for LC–MS/MS analysis [61, 62]. LC–MS/MS analysis included ExionLC™ AD UPLC and QTRAP® 6500+ MS/MS. For UPLC, Phase A/B used ultrapure water/acetonitrile containing 0.04% acetic acid and the injection volume was 2 μL. The gradient elution procedure was set as 0 min A/B of 95:5 (V/V), 1.0 min A/B of 95:5 (V/V), 8.0 min of 5:95 (V/V), 9.0 min of 5:95 (V/V), 9.1 min of 95:5 (V/V), 12.0 min of 95:5 (V/V) [63–65]. For MS/MS, the source temperature of ESI was 550°C, the mass spectrometry voltage was set to 5500 V/ –4500 V as positive/negative ion mode, and the CUR was set to 35 psi [66–68].

Upset figure, heat map and gene structure analysis used the TBtools software (www.tbtools.come). SNAC and C2H2-B subfamily proteins conserved domains were identified by NCBI CCD Tools, and their motifs were predicted by MEME which selected classic mode to find five motifs, multiple alignments used DNAMAN v8 (https://www.lynnon.com/), phylogenetic analysis using MEGA software with ML tree which was based on the JTT model, the bootstrap values were 200 bootstrap replicates. Microsoft Office (https://www.microsoft.com/zh-cn), R program and GraphPad Prism 8 were used to analyse the experimental data.

### Gene clone and plasmid construction

*PtNAC3* CDS regions and the 500 bp promoter regions of *PtZFP30* were amplified by PCR with the NEB Polymerase (#M0515), the primer listed in Table S1 (see online supplementary material). The plasmid pBI121, pHBT-GFP, pGBKT7, pGADT7, pB42AD, pLacZ2μ, pGEX-4 T-1, pGreenII 0800-LUC, and pGreenII 62-SK were obtained from Arabidopsis Biological Resource Center, and extracted by GoldHi EndoFree Plasmid Maxi kit (CoWin Biosciences; CW2104), Nanodrop 8000 was used to measure plasmids quality and quantity. The CDS of *PtNAC3* was recombined into the vector pBI121, pGBKT7 which used *OK Clon* DNA Ligation Kit (Accurate Biology; AG11802), and the CDS of *PtNAC3* was recombined into the vector pHBT-mCherry, pB42AD, pGEX-4 T-1 and pGreenII 62-SK; and the promoter of *PtZFP30* was recombined into the vector pLacZ2μ and pGreenII 0800-LUC by the protocol of BP Clonase II Enzyme mix (11789–020) and LR Clonase II Enzyme mix (11791–020).

### Subcellular localization analysis

The recombined plasmid pHBT-NAC3-mCherry was extracted using the CsCl gradient method to get purified plasmid, and the protocol was from the Sheen lab website. The protoplast transient expression assay followed the protocol as described previously [69, 70]. The 10 μL 20 mg/mL of DAPI was added into 1 mL W5 solution with transfected protoplast for 10 minutes to indicate nuclei. The confocal microscope (Leica SP8) was used to detect fluorescence signals (mCherry, excitation 587 nm, emission 600-620 nm; GFP, excitation 480 nm, emission 510-550 nm; DAPI, excitation 405 nm, emission 430-480 nm).

### Y1H assay and Y2H assay

The recombined plasmid pB42AD-*PtNAC3*, pGBKT7-*PtNAC3*, and pLacZ2μ-*PtZFP30p* were extracted by GoldHi EndoFree Plasmid Maxi kit (CoWin Biosciences; CW2104).

The recombined plasmid pB42AD-*PtNAC3* and pLacZ2μ-*PtZFP30p* were co-transformed into yeast EGY48 strains (Clontech; CAT#: YC1030) and grew on the SD-Trp/-Ura medium for 4 days at 28°C and transfer to SD/−Trp/-Ura + Gal+Raf + X-gal for 3 days at 28°C. The pLacZ2μ and the pB42AD, pLacZ2μ and the pB42AD-*PtNAC3*, the pLacz2μ-*PtZFP30p* and the pB42AD were used as the negative control.

The recombined plasmid pGBKT7-*PtNAC3* and pGADT7-T were co-transformed into yeast Y2HGold strains (Clontech; CAT#: YC1002) and grew on the SD-Leu/−Trp medium for 2 days at 30°C. The single colonies were transferred to SD-Trp-Leu-His-Ade medium for 5 days at 30°C.

### Effector/reporter-based luciferase transient expression assay

The recombined plasmid pGreenII 0800-LUC-*PtZFP30p* and pGreenII 62-SK-*PtNAC3* were extracted by GoldHi EndoFree Plasmid Maxi kit (CoWin Biosciences; CW2104); Nanodrop 8000 was used to measure plasmids quality and quantity.

The recombined plasmid pGreenII 0800-LUC-*PtZFP30p*, pGreenII 62-SK-*PtNAC3*, pGreenII 0800-LUC and pGreenII 62-SK were transformed into GV3101 *Agrobacterium* strain (Shanghai Weidi Biotechnology Co., Ltd; CAT#: AC1003) separately, the *Agrobacterium* GV3101 cells harbored the recombined plasmid were cultured overnight and transferred 1 mL *Agrobacterium* GV3101 cells into 20 mL LB broth (50 mg/L Kana., 50 mg/L Rif. and 15 μM acetosyringone) for approximately 10 hours at 28°C (OD600 = 0.5). The *Agrobacterium* GV3101 cells were then resuspended (OD600 = 1.0) by infiltration buffer with pH = 5.6 and containing 10 mM MgCl_2_, 10 mM MES, and 150 μM acetosyringone for 3 hours in the dark. Equal volumes of the suspensions were mixed and infiltrated into *N. benthamiana* leaves, the transgenic *N. benthamiana* were placed in the dark for 24 hours and then transferred to the greenhouse for 48 hours. Before fluorescence was detected by LB 983 NightOWL II (Berthold Technologies), leaves were sprayed with a solution containing 0.32 mg/mL D-Luciferin and 0.1% Triton X-100 (Sigma-Aldrich; 50 227).

### The electrophoretic mobility shift assay (EMSA)

The GST and PtNAC3-GST proteins were expressed by BL21(DE3) cells following the protocols [71]. The *PtZFP30* promoter probes containing ACACGTAA motifs (Table S1, see online supplementary material) which were 5′ biotin-labeled or unlabeled as control were synthesized by Beijing Tsingke. The EMSA luminous kit (GS009, Beyotime) was used to experiment. The images were taken by the BIORAD ChemiDocTM MP imaging system.

### CUT & Tag assay

The transient transformation assay in the hypocotyl of *P. tabuliformis* was carried out according to the method of Liu [72]. The cell nucleus extraction followed the step of CelLytic™ PN Isolation/Extraction Kit (CELLYTPN1; Sigma). The CUT & Tag assay was performed by NovoNGS CUT&Tag 3.0 High-Sensitivity Kit (Cat. No: N259-YH01) to construct the libraries. The droplet digital PCR (ddPCR) was performed with a 1 μl template in which CUT & Tag libraries were diluted five times. The ddPCR reagents were used from BioRad.

### The generation of transgenic lines

The recombined plasmid pBI121-PtNAC3 was transformed into *Agrobacterium* strain EHA105 (Shanghai Weidi Biotechnology Co., Ltd; CAT#: AC1010), and *35S::PtNAC3* overexpressed lines were generated by the floral-dip method [68, 73], and homozygous lines were screened by 50 mg/L Kana. MS mediums. Three *35S::PtNAC3* lines were used for further research, and 30 events were used to measure WT and transgenic lines’ lifespan.

## Acknowledgments

We thank Meiqin Liu and the Testing and Analysis Center of Beijing Forestry University for assisting in confocal microscopy analysis.

This work was supported by the Fundamental Research Funds for the Central Universities (NO. BLX202217, 2021BLRD22).

## Author contributions

S.N. conceived the research, participated in the design of the experiments, headed and managed the project and edited most of the manuscript. F.H. designed part of the experiments, carried out most of the experiments, analysed data and drafted the manuscript. P.W., X.C. and Y.N. planted *P. tabuliformis*, wild-type Arabidopsis, screened transgenic lines, and observed the phenotype, H.Z. and Q.Z. assisted with CUT & Tag assay, X.C. and Y.S. participated in analysing data. Y.L. and M.G. contributed to designing and supervising various parts of the research. All authors discussed and approved the final manuscript.

## Data availability

The authors declare that all data supporting the findings of this study are available within the article and supplementary material or are available upon request from the corresponding author. The RNA-seq data of drought treatment can be found at: http://db.cngb.org/search/project/CNP0002179; the RNA-seq data of UVB treatment can be found at: https://www.ncbi.nlm.nih.gov/bioproject/557580; the RNA-seq data of light treatment can found at: http://db.cngb.org/search/project/CNP0000737. Any additional information required to reanalyze the data reported in thiswork paper is available from the Corresponding Author upon request.

## Conflict of interest statement

None declared.

## Supplementary data

Supplementary data is available at *Horticulture Research* online.

## Supplementary Material

Web_Material_uhad130
