# Supplementary material for: An ethylene-induced NAC transcription factor acts as a multiple abiotic stress responsor in conifer
Source: Hortic Res. 2023 Jun 20;10(8):uhad130. doi: 10.1093/hr/uhad130 (PMC10407601; doi:10.1093/hr/uhad130)
Supplement: Web_Material_uhad130 [file web_material_uhad130.zip › Dataset S1.docx]

Dataset S1 The coding sequence of PtNAC3 and the promoter sequence of PtZFP30 in Chinese pine

>PtNAC3

ATGGGAAGACAGGATGCAGAGGCCCAGCTGAATTTACCGCCCGGATTCAGATTCTTCCCTACAGATGACGAGCTGGTAGTGCATTACTTGTGCAGGAAGGCTGCCTCACTGCCCATTGCTGTTCCTATTATTGCAGAGGTGGACTTGTACAAATTTGATCCATGGGAGCTGCCAGAAAAGGCACTGTTTGGAGAAAAGGAGTGGTATTTCTTCACTCCCAGGGACAGGAAGTATCCAAATGGTTCTCGCCCCAACAGGGCTGCTGGCTCAGGATACTGGAAAGCCACAGGTGCAGATAAACCCATCACTGCTAAGGGCAGCAACAGGCGTGTTGGCATCAAGAAGGCTCTGGTTTTCTATGTCGGAAAAGCACCTAAAGGAAACAAGACTAATTGGATTATGCATGAATACCGCCTTGCTGATGTCAATAGATCTGCAAAGAAGAAGGGCAGCTTAAGGTTGGATGATTGGGTACTTTGTCGAATATACAACAAGAAGAGCAGTGCGGAGAAGTTAGCTAAGGAGCAAGAGTGGTCCTCAGAAGAAGCAATGGAACAATTCCACGAAGAAATTGATCAAAAGGTGCCAGGAATACTGCCCACTGGGAATACTATAATGAACTCGAACATTGAGCATTCAGAAAGAATTTCACAAGATTCAACCAGATCTGCCCCCTCTCCGAATTGCAGAACAATCTCTAACCATGATTCAAGAACTTCTGCCATTACTTCCATGAGCTACAACTCAAACCCCATTTTTGAGCAGAATTTGAACATTTCAAATGCCAGTAGTGCTCCAATGGAGCTTCCAGAACTTGTCCCATTTTTCAACCCTATGACTAATCACAGGACAAATTACGATTCAGCAGATTTAATTCCTCCTATTCTGCTCACGGATTCAAGTTGTTCCATGCAATCGTCGCATGATCTTAAACCCGATAAAGAAGAAGTGCAGAGCAGCTGTAGATTGGAAGAACTGATGCAGCAACAGCGGCAGCAGCAGCAGGAGAATGCTGGTTTGAACCAATCAATGTTCACCTTTGGCTTCGAGAGTCTGCAAAACCCATTCCCGCAGTTAGACCAAATACAGCCTCCCTCCAGCAATGATCCTTTCCAAGATTACTTAGCCAGCCTCACAGCTCCAGGCTACTTACCGAGGTCTTCTTAT

>PtZFP30_promoter_

CCAATTGAATTAGAAGATGGTTGCCTACAAGTAATGCAGACACGCCTCCTCACACGTGGTATACTCAACCAAACCGAGTCAAGAGCTGAGTCGCGTGGTGGAGAAAACCAACGAGAAGTAGAAGACGAAGCATCGTGGAGAGTGTGGATTTTTGAGATTAGAGATTGACAGCGCCAACGCTGAGCAGACTGCTTCTTAATCAGCTTTTCACTTTGCAATCCAATCCAATATCCTATTCCACCCTTCACCCACCGCCACCCCGGAAGTCTGTTTTTCTGTACAATTTTGACCACTGGGCATCCTCTATACGTAAAATTCTGGTTACCCACCAGGAAGTATCGCGTGTTTTTTTAGACTTCTAGAAGCGAGGACTGAGCTCCCGGAGGCATCTGTTTTAAAGCTTGCTTTGAGCTTTCTTTTCCAGTAGTTAGTTATTTGATTCATATTGGTTGCAGAGGATTTTCAGAGTTTG
