# Supplementary material for: An ethylene-induced NAC transcription factor acts as a multiple abiotic stress responsor in conifer
Source: Hortic Res. 2023 Jun 20;10(8):uhad130. doi: 10.1093/hr/uhad130 (PMC10407601; doi:10.1093/hr/uhad130)
Supplement: Web_Material_uhad130 [file web_material_uhad130.zip › Supplemental figure.docx]

**Figure S1 The response profiles of global transcriptome and common induced genes under different abiotic stresses.**

(A) Upset diagrams displaying the number of abiotic stress response genes under different stress treatment. The number of genes induced by some abiotic stresses and repressed by other abiotic stresses did not exhibit in the diagrams. (B) The Dot plots showing the 32 signature abiotic stresses induced gene (ASIG) expressions across the five abiotic stresses clusters. The transcripts per million (TPM) were used to represent the gene expression level. The depth of the red dots represents expression levels of the ASIG.

**Figure S2 The expression pattern of ASITFs.**

(A) The first column represents the expression level of *PtNAC3* and *PtZFP30* under different temperature. 1-month-old *P. tabuliformis* seedlings were treated by five temperature gradients (4℃, 10℃, 20℃, 30 ℃ and 40℃) for 8 hours under long day (14h/10h) and the needles were harvested for RNA-seq; Data are shown as mean ±SEM (n=6). The second column represents the expression level of *PtNAC3* and *PtZFP30* under drought. Three-ear-old seedlings were treated without water for 8 or 23 days and then rewatered for 1 or 10 days then harvested the needles for RNA-seq; Data are shown as mean ±SEM (n=6). The third column represents the expression level of *PtNAC3* and *PtZFP30* under salt and osmotic treatment. 2-month-old seedlings were treated under long day (14h/10h) and irrigated 2M NaCl or 300mM mannitol once a day for 3 days, then harvested needles for RNA-seq. Data are shown as mean ±SEM (n=3). The fourth column represents the expression level of *PtNAC3* and *PtZFP30* under UVB. 2.5-year-old seedlings were irradiated by ultraviolet radiation for 1, 3 or 7 days in greenhouse before harvested for RNA-seq. Data are shown as mean±SEM (n=6). The fifth column represents the expression level of *PtNAC3* and *PtZFP30* under wound and cold. 2-month-old seedlings were cut off 1 cm and harvested needles after 8 hours, W&Cold represents the seedling were cut off 1 cm and move to 4 ℃ for 8 hours and harvested needles for RNA-seq. Data are shown as mean ±SEM (n=6). (B) The annual expression level of abiotic stress induced transcription factors (ASITF) in *P. tabuliformis* from 1st Jul. 2017 to 30th Dec. 2018 in Beijing. The X-axis indicates the sampling date from July 1st 2017 to July 2nd 2018, the needles were collected from three individual trees at eleven O'clock. The date was visualized by GraphPad. Data are shown as mean ±SEM (n=3). (C) The co-expression relativity between *PtNAC3* and ASITFs in *P. tabuliformis*. The transcript date of ASITFs shown in tableS2 under abiotic stress treatment were used to analyzed correlation coefficients. GraphPad was used to calculate the correlation coefficients with default parameters. (D) The daily expression level of ASITFs in adult *P. tabuliformis* from 1st Jul. 2017 to 30th Dec. 2018 in Beijing. The needles were collected from three individual trees at eleven O'clock. The date was visualized by GraphPad. Data are shown as mean ±SEM (n=3).

**Figure S3 PtNAC3 is a conserved stress-related NAC (SNAC) transcription factor.**

Maximum likelihood phylogenetic tree of NAC family proteins in plants. The red bar outside the IDs denotes *P. tabuliformis* homolog identified in the study. The full-length protein sequences from *Chlamydomonas reinhardtii* (green algae), *Marchantia polymorpha* (liverwort), *Selaginella moellendorffii* (selaginella), *Physcomitrella patens* (moss), *A. thaliana* (herbaceous angiosperm), *Populus trichocarpa* (woody angiosperm) were used to build the tree. The supporting values are shown on the branches in the following order: SH-aLRT test/bootstrap value. The phylogenetic tree was constructed using the maximum likelihood Jones-Taylor-Thornton (JTT) algorithm model. The percentages of the bootstrap consensus tree which was inferred from 1000 replicates were marked out next to the branches. Evolutionary analyses were conducted in MEGA-X.

**Figure S4 The expression level of *PtNAC3* in different *35S::PtNAC3* transgenic lines.**

Three events of *35S::PtNAC3*(OE1-OE3) and wildtype(WT) *Arabidopsis* were randomly selected for semi-quantitative PCR. 50 μL reaction system were used including 1 μg RNA reverse transcription of cDNA, 0.2 μM primers and DNA Polymerase Mix (B2241JAD, Vazyme), primers used in the study were listed in Table S1 and labeled on top of the agarose gel plot, the extracted RNA of the used plant material is labeled on the right side of the agarose gel plot.


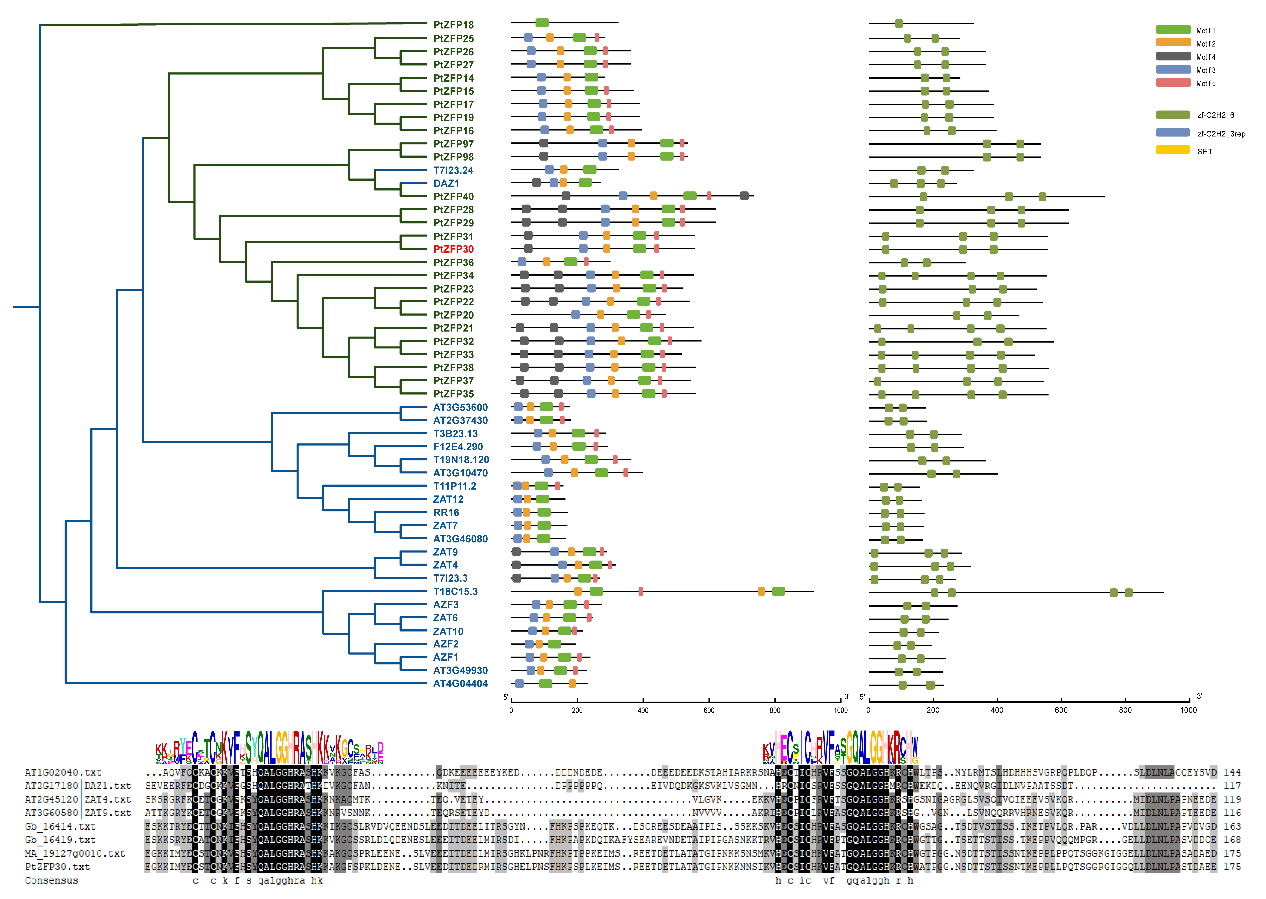


**Figure S5 The sequence conservation analysis of *PtZFP30*.**

Phylogenetic analysis of the motif pattern and conserved domain analysis of C2H2 family proteins. The full-length protein sequences from *A. thaliana* and *P. tabuliformis* were used to build the tree. The supporting values are shown on the branches in the following order: SH-aLRT test/bootstrap value. The phylogenetic tree was constructed using the maximum likelihood Jones-Taylor-Thornton (JTT) algorithm model. The percentages of the bootstrap consensus tree which was inferred from 1000 replicates were marked out next to the branches. Evolutionary analyses were conducted in MEGA-X.

**Figure S6 Expression patterns of *PtNAC3* and *PtZFP30* in tissues of different parts of *Pinus tabuliformis***

The large-scale RNA-seq data from 760 biological samples to reflect the expression patterns of PtNAC3 and PtZFP30 in *Pinus tabuliformis*.

**
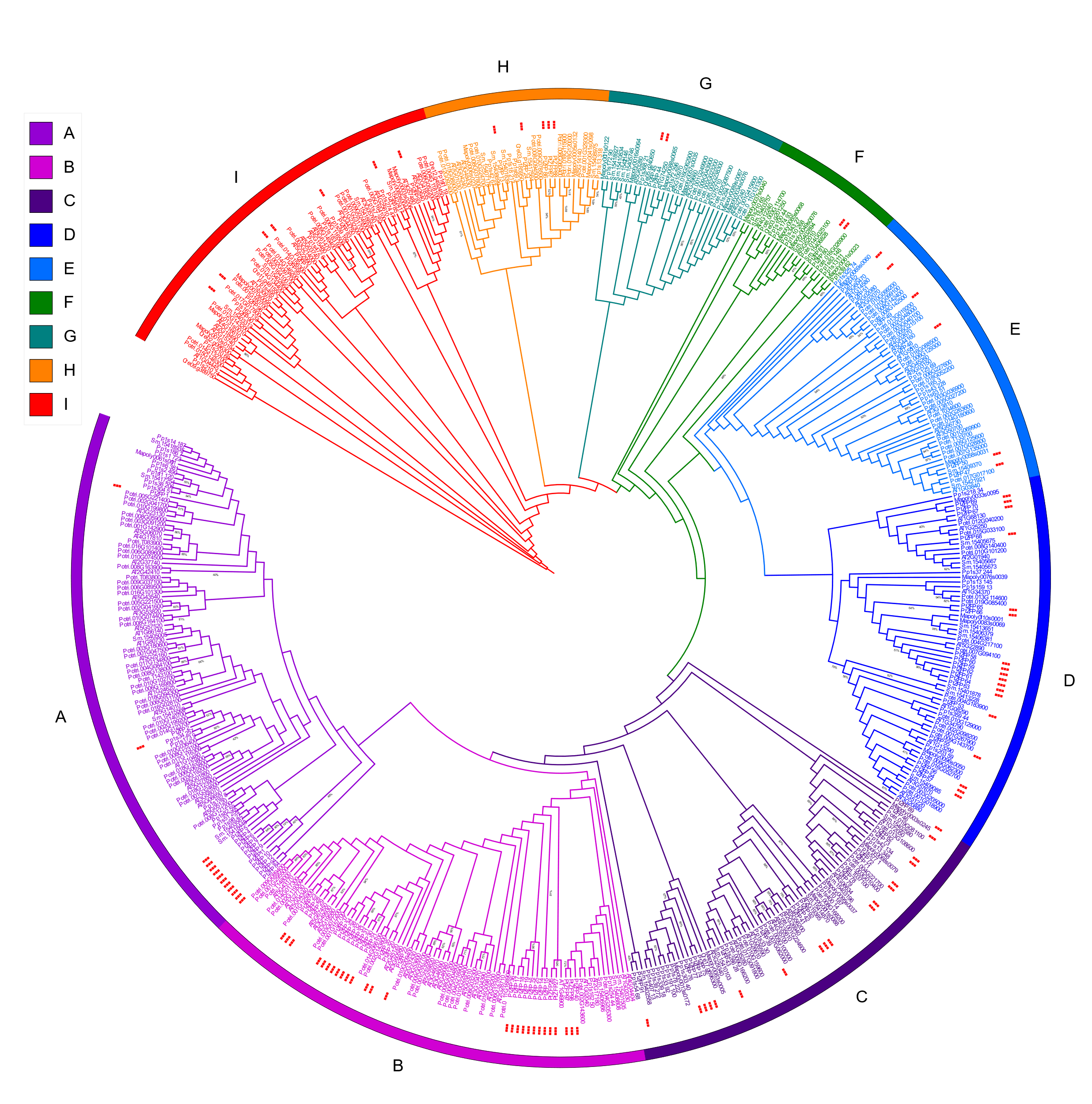
**

**Figure S7 PtZFP30 is a C2H2 transcription factor.**

Maximum likelihood phylogenetic tree of C2H2 family proteins in plants. The red bar outside the IDs denotes *P. tabuliformis* homolog identified in the study. The full-length protein sequences from *Chlamydomonas reinhardtii* (green algae), *Marchantia polymorpha* (liverwort), *Selaginella moellendorffii* (selaginella), *Physcomitrella patens* (moss), *A. thaliana* (herbaceous angiosperm), *Populus trichocarpa* (woody angiosperm) were used to build the tree. The supporting values are shown on the branches in the following order: SH-aLRT test/bootstrap value. The phylogenetic tree was constructed using the maximum likelihood Jones-Taylor-Thornton (JTT) algorithm model. The percentages of the bootstrap consensus tree which was inferred from 1000 replicates were marked out next to the branches. Evolutionary analyses were conducted in MEGA-X.

**Table S1 Primers used in qRT-PCR analysis and plasmid construction**

| **qPCR primers** | |
| --- | --- |
| **Primer name** | **Primer sequence（5‘-3’）** |
| Tubulin-F | GGCATACCGGCAGCTCTTC |
| Tubulin-R | AAGTTGTTGGCGGCGTCTT |
| PtNAC3-F | TCCTTTGGCTTCGAGAGTC |
| PtNAC3-R | TAAGTAGCCTGGAGCTGTG |
| ZFP30-F | CCAATTGAATTAGAAGATGGTTGCC |
| ZFP30-R | CAAACTCTGAAAATCCTCTGCAAC |
| **Plasmid construction primers** | |
| PtNAC3-F | ATGGGAAGACAGGATGCAGAG |
| PtNAC3-R | ATAAGAAGACCTCGGTAAGTAGCCT |
| PBI121-PtNAC3-F | CACGGGGGACTCTAGATGGGAAGACAGGATGCAGAGG |
| PBI121-PtNAC3-R | CCCTTGCTCACCATGATAAGAAGACCTCGGTAAGTAGCC |
| PGBKT7-PtNAC3-F | CATGGAGGCCGAATTCATGGGAAGACAGGATGCAGAG |
| PGBKT7-PtNAC3-R | GCCGCTGCAGGTCGACCTAATAAGAAGACCTCGGTAAGTAG |
| gateway-PtNAC3-F | GGGGACAAGTTTGTACAAAAAAGCAGGCTTTATGGGAAGACAGGATGCAGAG |
| gateway-PtNAC3-R | GGGGACCACTTTGTACAAGAAAGCTGGGTTATAAGAAGACCTCGGTAAGTAGCCT |
| gateway-PtZFP30_promoter_-F | GGGGACAAGTTTGTACAAAAAAGCAGGCTTTCCAATTGAATTAGAAGATGGTTGCC |
| gateway-PtZFP30_promoter_-R | GGGGACCACTTTGTACAAGAAAGCTGGGTTCAAACTCTGAAAATCCTCTGCAACC |
| **EMSA probe** | |
| ZFP30p-F | CATCCTCTATACGTAAAATTCTGGTTACCCACCAGGAAGTATCGCGTGTTT |
| ZFP30p-R | AAACACGCGATACTTCCTGGTGGGTAACCAGAATTTTACGTATAGAGGATG |
